# Research hotspots and trends of microRNAs in intervertebral disc degeneration: a comprehensive bibliometric analysis

**DOI:** 10.1186/s13018-023-03788-4

**Published:** 2023-04-15

**Authors:** Shuang Chen, Yi Wang, Huanxi Wu, Xiaoyang Fang, Chenyu Wang, Nan Wang, Lin Xie

**Affiliations:** 1 Affiliated Hospital of Integrated Traditional Chinese and Western Medicine, Nanjing University of Chinese Medicine, Nanjing, China; 2 The Second Clinical Medical College, Nanjing University of Chinese Medicine, Nanjing, China

**Keywords:** Intervertebral disc degeneration, MicroRNAs, Bibliometrics, Apoptosis, Inflammation, Proliferation, Degradation

## Abstract

**Background:**

MicroRNAs (miRNAs) are involved in various pathological processes, such as proliferation, growth, and apoptosis, of intervertebral disc (IVD) cells and play an important role in the development of intervertebral disc degeneration (IDD). Although some studies have reported the role of miRNAs in IDD, scientific econometric analysis in this field is not available.

**Objectives:**

We designed this study to describe the current research trends and potential mechanisms associated with the role of miRNAs in IDD and to provide new ideas for future research in this field.

**Methods:**

We conducted a bibliometric analysis of the publications on the role of miRNAs in IDD included in the Web of Science core collection database to elucidate the current research trends in this field. The potential mechanisms were constructed using the Arrowsmith project.

**Results:**

We found that the number of miRNAs and IDD-related publications increased over the years. China was the most important contributor to research in this field. The top three institutions in terms of number of articles published were Huazhong University of Science and Technology, Shanghai Jiao Tong University, and Xi’an Jiao Tong University. Shanghai Jiao Tong University had the highest number of citations. *Experimental and thermal medicine* had the maximum number of documents, and *Cell promotion* had the most citations. The journal with the most mean times cited per study was *Annals of the Rheumatic Diseases*. The author Wang K had the highest number of publications, and Wang HQ had the highest number of citations. These two authors made important contributions to the research in this field. The keyword analysis showed that recent studies have focused on miRNAs regulating nucleus pulposus cell apoptosis and proliferation. Moreover, we revealed the potential mechanisms of miRNAs associated with IDD, including miRNAs regulating the extracellular matrix (ECM) degradation, mediating cartilage endplate (CEP) degeneration, and participating in inflammatory responses.

**Conclusion:**

We demonstrated the knowledge map of miRNAs and IDD-related research through bibliometric analysis and elucidated the current research status and hotspots in this field. The mechanisms by which miRNAs regulate the apoptosis and proliferation of degenerated IVDs, promote ECM degradation, mediate CEP degeneration, and participate in inflammatory responses should be explored in further studies.

**Supplementary Information:**

The online version contains supplementary material available at 10.1186/s13018-023-03788-4.

## Introduction

Intervertebral disc degeneration (IDD) can cause pathological changes, such as spinal stenosis, spinal segmental instability, osteophytes, and nerve root compression, which are the main causes of cervical and low back pain (LBP) [1], which, in turn, reduces the quality of life and work efficiency of patients and brings huge economic costs to the society [2, 3]. IDD is associated with inflammatory mediators, abnormal metabolism of extracellular matrix (ECM), epigenetic changes of normal intervertebral discs (IVDs), decrease in the active cells, and increase in the senescent cells [4, 5]; however, its pathological mechanism remains unclear. Currently, several therapeutic methods are used for degenerative disc disease (DDD); nevertheless, they are limited to symptomatic management (conservative treatments and surgical interventions), which cannot delay or reverse the pathological process of IDD. Therefore, studying the pathogenesis of IDD at the molecular level, halting or reversing the resulting pathological changes at the cellular level, and restoring the structure and function of the disc are current challenges and areas of active research in global orthopaedic research [6].

MicroRNAs (miRNAs) are a type of endogenous noncoding single-stranded RNA molecules with 20–25 nucleotides, and they widely exist in eukaryotic cells. Although they account for only 1–3% of the human genome, they can regulate approximately 30% of the protein-coding genes [7]. MiRNAs can regulate protein expression by shearing, inhibiting, or enhancing the translation of target mRNA, thereby participating in various pathophysiological processes, such as cell proliferation, growth, differentiation, and apoptosis [8–10]. MiRNAs play an important role in the occurrence and development of tendon injuries, osteoarthritis, rheumatoid arthritis, IDD, and other diseases [11–13]. Although some studies have reported the role of miRNAs in IDD [14, 15], scientific econometric analysis in this field has not been reported.

Bibliometric analysis quantitatively compares the academic contributions of different countries/regions, institutions, authors, and journals in a specific research field using various metrics derived from the literature. It utilizes visualization techniques to display the relevant specific information and development trends in the research field [16]. VOSviewer developed by Van Eck [17] from Leiden University, the Netherlands, and CiteSpace developed by Chen [18] from Drexel University in the United States are the two commonly used bibliometric softwares for analysing the current status of disciplinary research, identifying research hotspots and trends, and determining research directions. We conducted a bibliometric analysis of studies related to miRNAs and IDD to draw a visual knowledge map and provide a comprehensive description of the research hotspots, trends, and potential mechanisms associated with miRNAs and IDD to offer new ideas and approaches for the treatment of IDD.

## Materials and methods

### Data sources and search strategies

We searched the Web of Science core collection (WOSCC) database covering all available data up to 30 November 2022. The publication type was limited to “article” and “review”, and the language was restricted to English to extract articles of high quality. All searches were completed on 30 November 2022 and imported into the bibliometric tools for analysis to avoid bias caused by database updates. The search strategy was: TS = ((mi*rna* OR mir)) AND TS = ((“Intervertebral Disc Degeneration*”) OR (“Degeneration, Intervertebral Disc*”) OR (“Disc Degeneration, Intervertebral*”) OR (“Intervertebral Disc Degenerations*”) OR (“Disc Degeneration*”) OR (“Degeneration, Disc*”) OR (“Disc Degenerations*”) OR (“Intervertebral Disk Degeneration*”) OR (“disc hernias*”) OR (“disc disease*”) OR (“disk disease*”) OR (“disk hernias*”)). Two researchers independently screened the articles, and the third researcher was responsible for the review. The three researchers negotiated to solve the problem in case of disagreement. The document screening process is shown in Additional file 1: Fig. S1.

### Data collection

The search results were downloaded in “Plain text file” format with full records and cited references in the WOSCC database. After careful screening, the data were imported into Microsoft Excel 2016 and bibliometric softwares for further analysis.

### Bibliometric analysis

We used the Microsoft Excel 2016 software to collect the general information including the number of publications, countries/regions, institutions, journals, and authors. The impact factor (IF) and partition of the journals were based on the data of the 2020 Journal Ranking by Clarity Analytics Journal Citation Reports (JCR). We used VOSviewer 1.6.18 and Scimago Graphica for visual analysis of the data, including countries/regions, institutions, core journals, active authors, co-cited references, and keyword clustering analysis. CiteSpace 5.8.R1 was used to graph country/region co-occurrence networks, dual-map overlay of journals, and perform keyword emergence analysis to identify research hotspots and trends over a certain time. In addition, we used the *R* software “Biblimetrix Package” for the thematic evolution of keywords with the time cut-off points set at 2020 and 2022 (3 years and 1 year).

### Analysis of the potential mechanisms based on the Arrowsmith project

We used the Arrowsmith project (http://arrowsmith.psych.uic.edu) to construct the relationship between miRNAs and IDD and explore the potential mechanisms of miRNAs in IDD [19]. First, the keywords obtained from the Arrowsmith project were used as the prediction group, and the keywords extracted from VOSviewer were used as the confirmation group. A Venn diagram of the two groups was drawn to obtain potential keywords for miRNAs and IDD-related research. We used the *R* software for Gene Ontology (GO) analysis and Kyoto Encyclopedia of Genes and Genomes (KEGG) pathway analysis of the potential genes (correlation probabilities ≥ 0.95). The protein–protein interaction (PPI) network was constructed and analysed for the potential genes using the STRING online database (https://www.stringdb.org/) and visualized in the Cytoscape software. In addition, the top 10 hub genes in the PPI network were screened using the CytoHubba plugin of the Cytoscape software using the maximal clique centrality (MCC) method.

## Results

### Annual publications and trends

**Fig. 1:**
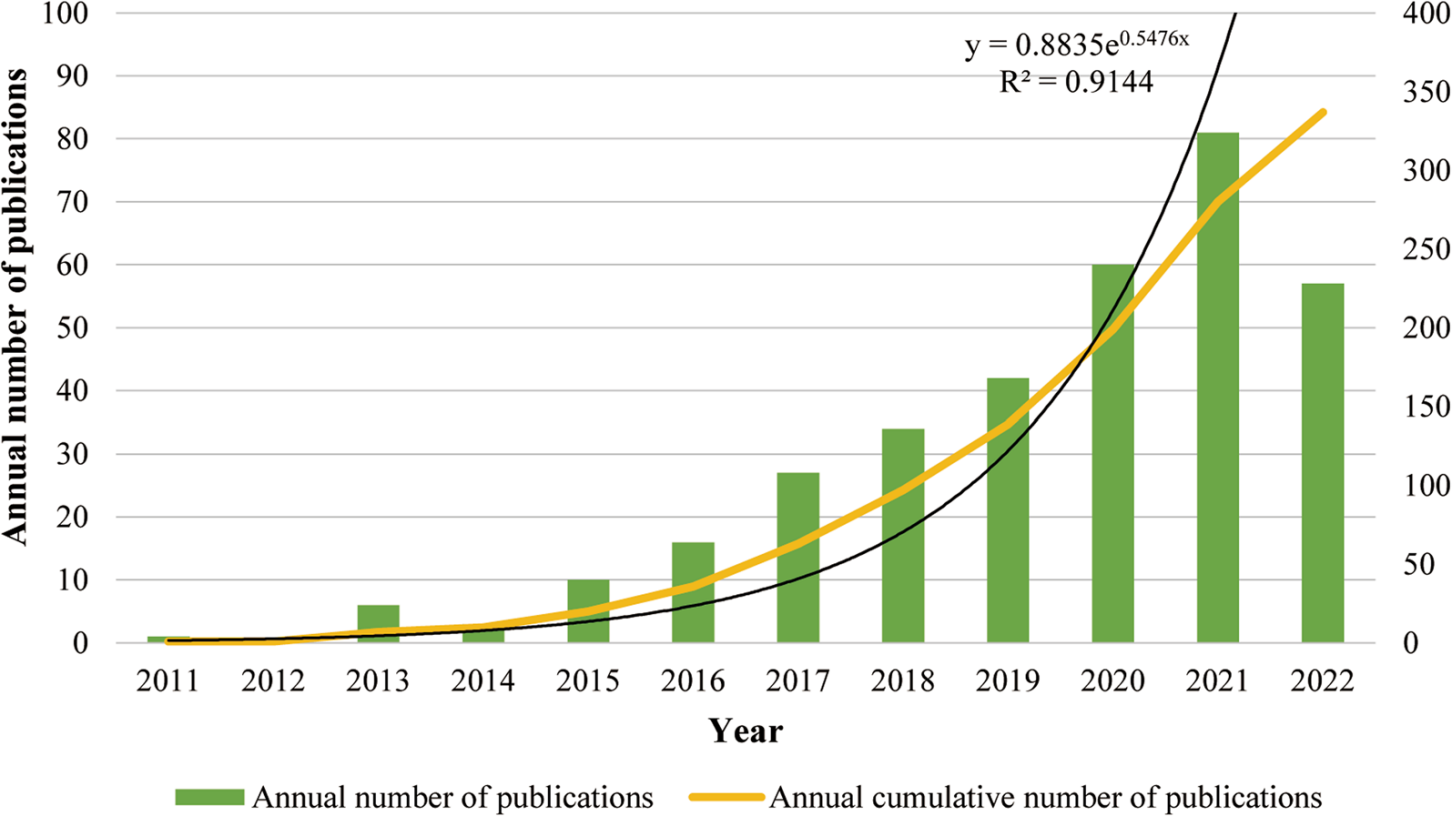
Annual and cumulative number of publications on miRNAs in IDD research

We finally included 337 articles in this study. The annual number of publications showed an overall upward trend, but the number of papers published in 2022 dropped, which may be because data were limited to 30 November 2022 (Fig. 1). The annual cumulative number of published papers was fitted by a polynomial function, and the fitting curve function formula was “*y* = 0.8835e^0.5476x^, *R*^2^ = 0.9144”, indicating a good fit of the research growth curve in this field. These results indicated that the study of miRNAs may be a trending topic in IDD.

### Country/region analysis and international cooperation

**Fig. 2:**
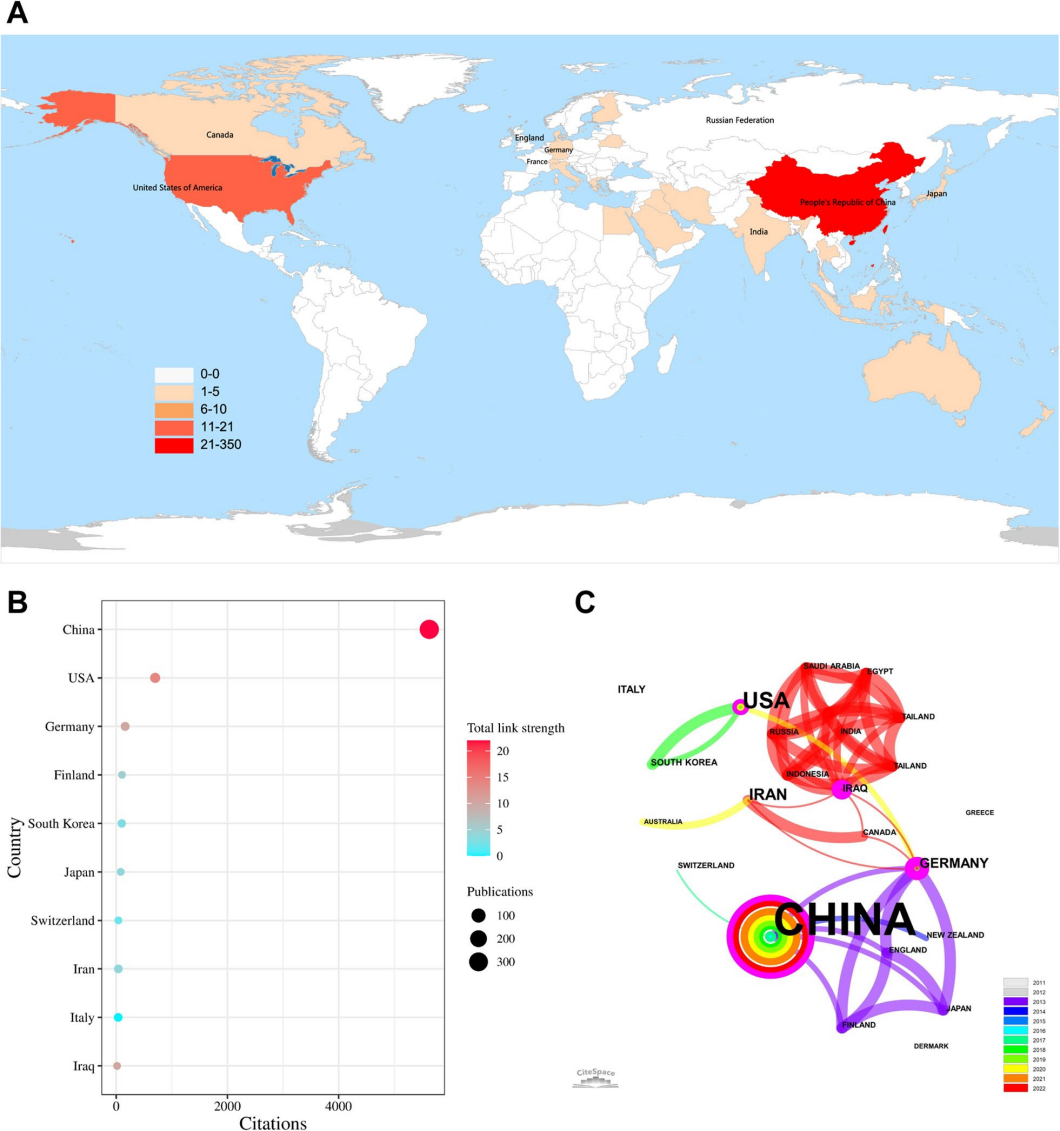
National/regional contributions of miRNAs and IDD-related research. **A** World map of the publications in the WOSCC database. The intensity of the red colour is proportional to the number of publications. The China board on the world map has the highest intensity of red colour, indicating that the maximum number of documents are published from this region. **B** Bubble chart of citation frequency of countries/regions. **C** National/regional cooperation network. The node represents the country/region, the size of the node represents its documents, and the colour of the connection between nodes represents the year of the first cooperation between nodes

We found that the literature in this field was published from 23 countries/regions, and Fig. 2A shows the global distribution of publications. Figure 2B shows that more than four-fifths of documents are from China (*n* = 320; 86.02%) followed by the United States (*n* = 15; 4.03%) (Table 1). China has the highest citation frequency (*n* = 5632; 77.94%) followed by the United States (*n* = 700; 9.69%) and Germany (*n* = 160; 2.21%). The international cooperation network mapped by the CiteSpace software is presented in Fig. 2C. The countries/regions that closely cooperated with China were mainly distributed in Europe, including Germany, England, and Finland.

**Table 1:** Top 10 countries/regions in terms of the number of documents and citations on miRNAs in IDD

Rank	Country	Documents (%)	Rank	Country	Citations (%)
1	China	320 (86.02)	1	China	5632 (77.94)
2	The United States	15 (4.03)	2	The United States	700 (9.69)
3	Germany	5 (1.34)	3	Germany	160 (2.21)
4	Iran	4 (1.08)	4	Finland	104 (1.44)
5	Italy	4 (1.08)	5	South Korea	99 (1.37)
6	South Korea	3 (0.81)	6	New Zealand	83 (1.15)
7	Finland	2 (0.54)	7	Japan	80 (1.11)
8	Iraq	2 (0.54)	8	England	75 (1.04)
9	Japan	2 (0.54)	9	Switzerland	39 (0.54)
10	Switzerland	2 (0.54)	10	Iran	38 (0.53)

*miRNAs* microRNAs; *IDD* Intervertebral disc degeneration

### Institutional contribution

**Fig. 3:**
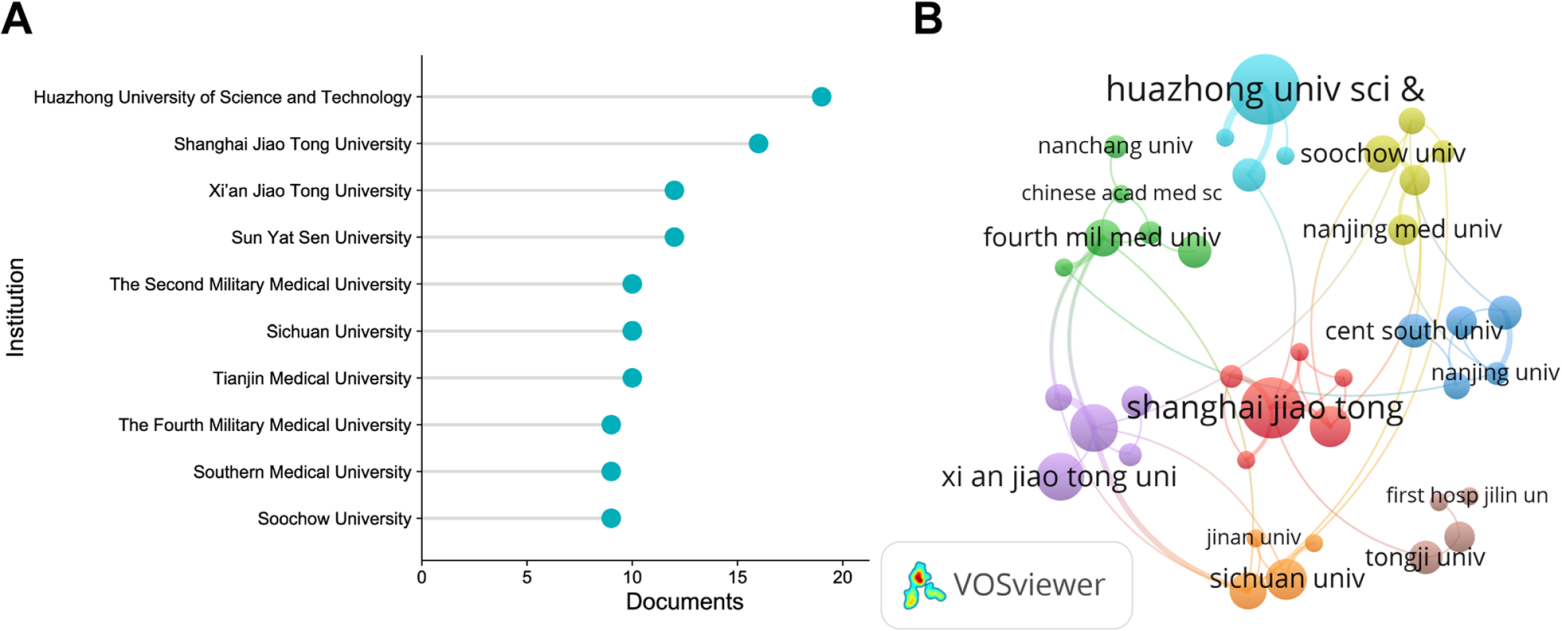
Institutional contribution on miRNAs in IDD research. **A** Lollipop chart of the top 10 institutions in terms of the number of documents. **B** Network map of the contributions and collaborations of the core institutions. The nodes indicate institutions with larger nodes indicating a larger number of documents. Different colours represent different clusters

Three hundred and fifty-seven institutions have published literature in this field. The top 10 institutions in terms of the number of documents and citations were from China (Table 2). The top 3 institutions in terms of the number of publications were Huazhong University of Science and Technology (*n* = 19; 2.79%), Shanghai Jiao Tong University (*n* = 16; 2.35%), and Xi’an Jiao Tong University (*n* = 12; 1.76%) (Fig. 3A). Shanghai Jiao Tong University (*n* = 498; 3.38%) had the highest citation frequency.

**Table 2:** Top 10 institutions in terms of the number of documents and citations on miRNAs in IDD

Rank	Institution (*n* = 357)	Documents (%)	Rank	Institution (*n* = 357)	Citations (%)
1	Huazhong University of Science and Technology	19 (2.79)	1	Shanghai Jiao Tong University	498 (3.38)
2	Shanghai Jiao Tong University	16 (2.35)	2	The Fourth Military Medical University	439 (2.98)
3	Xi’an Jiao Tong University	12 (1.76)	3	Huazhong University of Science and Technology	430 (2.92)
4	Sun Yat-sen University	12 (1.76)	4	The University of Hong Kong	356 (2.42)
5	The Second Military Medical University	10 (1.47)	5	Shanghai Municipal Center for Disease Control and Prevention	335 (2.27)
6	Sichuan University	10 (1.47)	6	University of Rochester	335 (2.27)
7	Tianjin Medical University	10 (1.47)	7	Yangzhou University	335 (2.27)
8	The Fourth Military Medical University	9 (1.32)	8	Xi’an Jiao Tong University	304 (2.06)
9	Southern Medical University	9 (1.32)	9	Nanjing University	299 (2.03)
10	Soochow University	9 (1.32)	10	The Chinese University of Hong Kong	284 (1.93)

*miRNAs* microRNAs; *IDD* Intervertebral disc degeneration

The core institutions were determined according to Price’s law ($N = 0.749\sqrt {\max }$, where max represented the number of publications issued by the institution with the highest number of documents, and an institution with documents > *N* was a core institution [20]). The VOSviewer software was used to visualize and analyse the cooperation of the core institutions. The core institutions were divided into eight clusters (Fig. 3B). Shanghai Jiao Tong University had the highest total link strength and formed cluster 1 with The Second Military Medical University. Xi’an Jiao Tong University and Sun Yat-sen University were grouped into cluster 5, and Huazhong University of Science and Technology belonged to cluster 6. Overall, the cooperation between the core institutions with more publications was still not extensive.

### Journal analysis

**Fig. 4:**
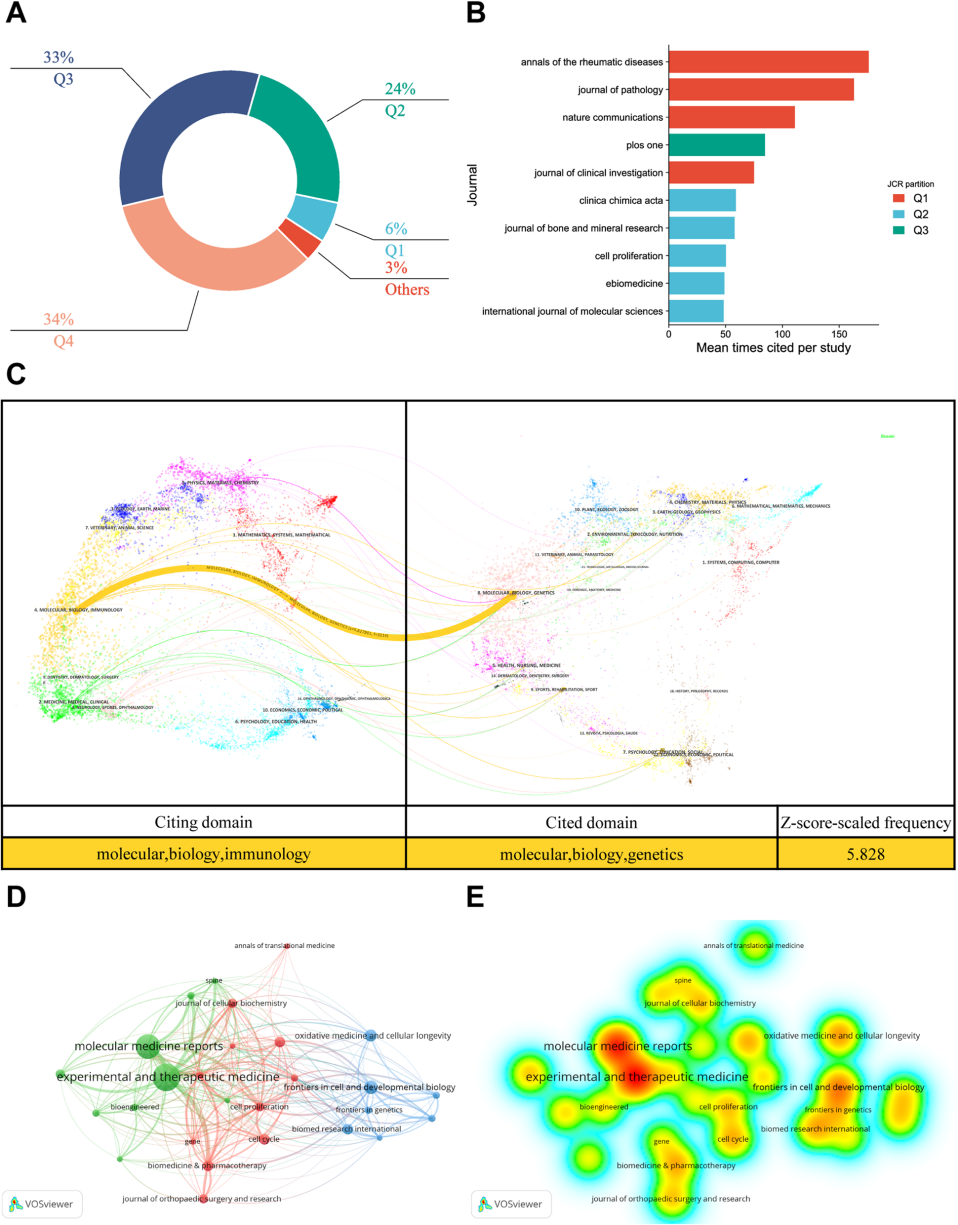
Journal characteristics of miRNAs and IDD-related research. **A** JCR partition of the journals on miRNAs and IDD-related research. **B** Bar chart of the top 10 journals in terms of the mean times cited per study. **C** Dual-map overlay of journals in the field. The dual-map overlay of journals in the field showed one main citation path in yellow. **D** Visual network map of core journals. Different colours represent different clusters. The nodes indicate journals, and the larger the node, the more publications in the journal. **E** Visual density view of the core journals. The node density size depends on the number of elements in the surrounding area and the importance of these elements. The higher the node density and closer to red, the more articles are published. Conversely, the lower the density and closer to blue, the fewer articles are published

The relevant literature was published in 151 journals. According to the JCR data of 2020, 9 journals (6%) appeared in the Q1 partition and 36 journals (24%) in the Q2 partition (Fig. 4A), thereby revealing that the research on miRNAs in IDD was still in its initial stage and the research quality still needed to be further improved. *Experimental and therapeutic medicine* (*n* = 18; 5.34%) had the most publications in this field. *Cell proliferation*, *Journal of cellular and molecular medicine*, and *Plos one* were the three most cited journals (Additional file 1: Table S1). *Annals of the Rheumatic Diseases*, *The Journal of Pathology*, and *Nature Communications* had the most mean times citations per study, and they belonged to the Q1 partition. Therefore, these journals may be more authoritative in this field (Fig. 4B).

In the dual-map overlay of journals, the left side of the curve represented the references cited in the study, whereas the right side of the curve represented the cited reference source (Fig. 4C). We found that the miRNAs and IDD-related publications had one main citation path. The citation path showed that the literature in the field of “molecular, biology, immunology” mainly referred to the literature published in the field of “molecular, biology, genetics”. According to Price’s law, the journal with a number of publications ≥ 4 was the core journal (*N* = 3.18). The cooperative network map showed that the core journals were divided into three clusters, and *Experimental and therapeutic medicine* and *Molecular medicine reports* in cluster 1 had the largest node/density in the network, thereby indicating that these journals played an important role in this field (Fig. 4D, E).

### Author contribution and collaboration analysis

**Fig. 5:**
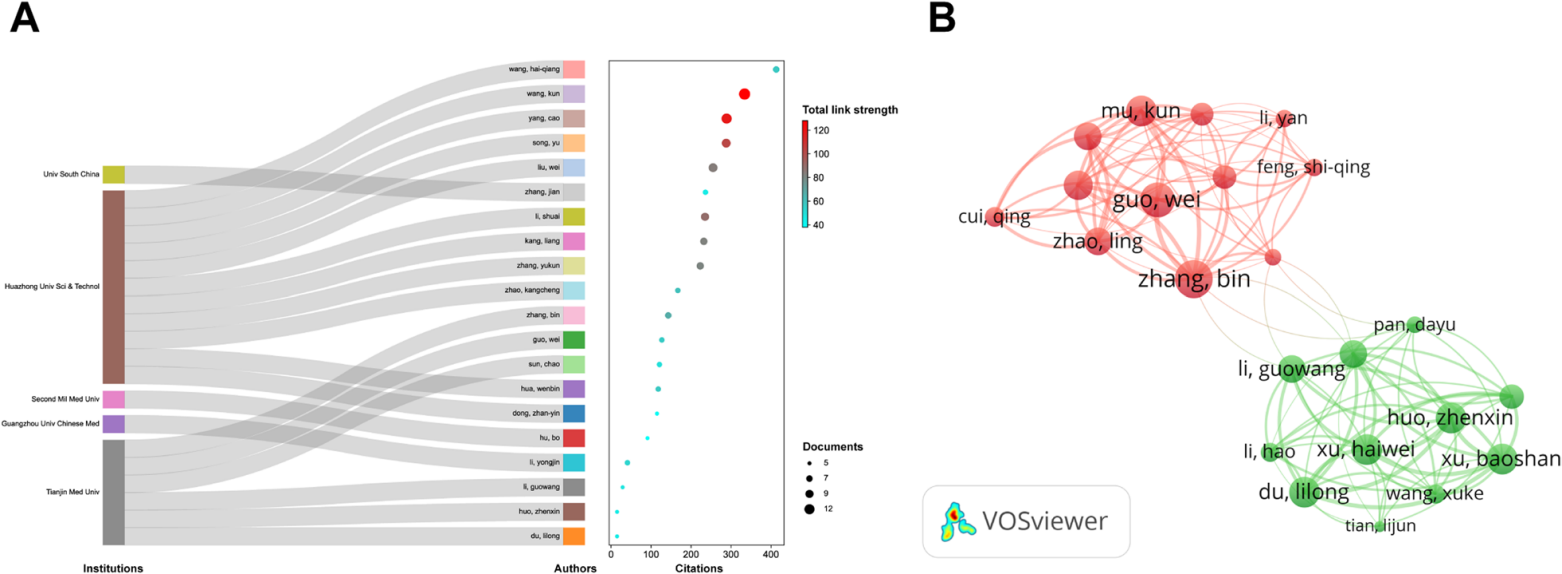
Core author characteristics of miRNAs and IDD. **A** Sankey dot plot of the top 20 authors with their institutions in terms of the number of publications. **B** Network map of contributions and collaborations of the core authors. Different colours represent different clusters. Each node represents an author. The size of the node is directly proportional to the importance of the author in the network

A total of 1646 authors have published relevant literature in this field. The top 10 authors were from China, and they published 9.02% of the literature in this field (Table 3). The two authors who published the most papers were Wang K (*n* = 14; 1.42%) and Yang C (*n* = 12; 1.21%). Wang HQ had the most citations (*n* = 413; 1.67%) followed by Cheng XF and Hu Y. Figure 5A shows the association between the top 20 authors and their institutions. The results showed that most of the high-productivity authors were from the Huazhong University of Science and Technology, indicating that they extensively studied the pathogenesis of IDD at the molecular level.

**Table 3:** Top 10 authors in terms of the number of documents and citations on miRNAs in IDD

Rank	Author (*n* = 1646)	Documents (%)	Total link strength	Rank	Author (*n* = 1646)	Citations (%)	Total link strength
1	Wang K	14 (1.42)	101	1	Wang HQ	413 (1.67)	38
2	Yang C	12 (1.21)	95	2	Cheng XF	335 (1.36)	18
3	Song Y	10 (1.01)	89	3	Hu Y	335 (1.36)	18
4	Liu W	10 (1.01)	55	4	Li H	335 (1.36)	18
5	Li S	9 (0.91)	77	5	Li YM	335 (1.36)	18
6	Zhang YK	8 (0.81)	75	6	Sun XJ	335 (1.36)	18
7	Kang L	8 (0.81)	69	7	Zhang GY	335 (1.36)	18
8	Zhang B	7 (0.71)	55	8	Zhang K	335 (1.36)	18
9	Wang HQ	7 (0.71)	38	9	Zhang L	335 (1.36)	18
10	Zhao KC	6 (0.61)	55	10	Zhao CQ	335 (1.36)	18

*miRNAs* microRNAs; *IDD* Intervertebral disc degeneration

We used the VOSviewer software to visualize and analyse the collaboration of the authors. According to Price’s law, authors with publications ≥ 3 were identified as the core authors (*N* = 2.80), and 123 authors with documents ≥ 3 were divided into two clusters, indicating close cooperation among the core authors (Fig. 5B).

### Analysis of highly cited articles and co-citation articles

The top 10 publications in terms of the number of citations are listed in Table 4. Among them, an article published by Cheng X [21] in *Annals of the Rheumatic Diseases* in 2018 was the most cited article with 176 citations. This study reported that circVMA21 alleviated nucleus pulposus cell (NPC) apoptosis and ECM synthesis catabolic imbalance induced by inflammatory cytokines through the miR-200c-XIAP pathway. Wang HQ [22] published the article entitled “Deregulated miR-155 promotes Fas-mediated apoptosis in human intervertebral disc degeneration by targeting FADD and caspase-3” in *The Journal of Pathology* in 2011, which was cited 163 times and ranked second in terms of citations. The authors concluded that the regulated miR-155 promoted Fas-mediated apoptosis in human IVDs by targeting FADD and caspase-3. We found that 40% of the top 10 highly cited publications were studies on the mechanisms of NPC apoptosis from the perspective of miRNAs [21–24], thereby suggesting that apoptosis played a crucial role in maintaining the stability of IVD.

**Table 4:** Top 10 highly cited articles on miRNAs in IDD in the WOSCC database

Rank	Author	Title	Journal	Year	Citations
1	Cheng X	Circular RNA VMA21 protects against intervertebral disc degeneration through targeting miR-200c and X-linked inhibitor-of-apoptosis protein	*Annals of the Rheumatic Diseases*	2018	176
2	Wang HQ	Deregulated miR-155 promotes Fas-mediated apoptosis in human intervertebral disc degeneration by targeting FADD and caspase-3	*The Journal of Pathology*	2011	163
3	Cheng X	Mesenchymal stem cells deliver exogenous miR-21 via exosomes to inhibit nucleus pulposus cell apoptosis and reduce intervertebral disc degeneration	*Journal of Cellular and Molecular Medicine*	2018	159
4	Yu X	MicroRNA-10b Promotes Nucleus Pulposus Cell Proliferation through RhoC-Akt Pathway by Targeting HOXD10 in Intervertebral Disc Degeneration	*PloS One*	2013	150
5	Li Z	MicroRNA in intervertebral disc degeneration	*Cell Proliferation*	2015	134
6	Ji ML	Preclinical development of a microRNA-based therapy for intervertebral disc degeneration	*Nature Communications*	2018	111
7	Fang H	miRNA-21 promotes proliferation and invasion of triple-negative breast cancer cells through targeting PTEN	*American Journal of Translational Research*	2017	96
8	Wan ZY	Aberrantly expressed long noncoding RNAs in human intervertebral disc degeneration: a microarray related study	*Arthritis Research & Therapy*	2014	83
9	Gu SX	MicroRNA-146a reduces IL-1-dependent inflammatory responses in the intervertebral disc	*Gene*	2015	80
10	Chen WK	lncRNAs: novel players in intervertebral disc degeneration and osteoarthritis	*Cell Proliferation*	2017	79

*miRNAs* microRNAs; *IDD* Intervertebral disc degeneration

**Fig. 6:**
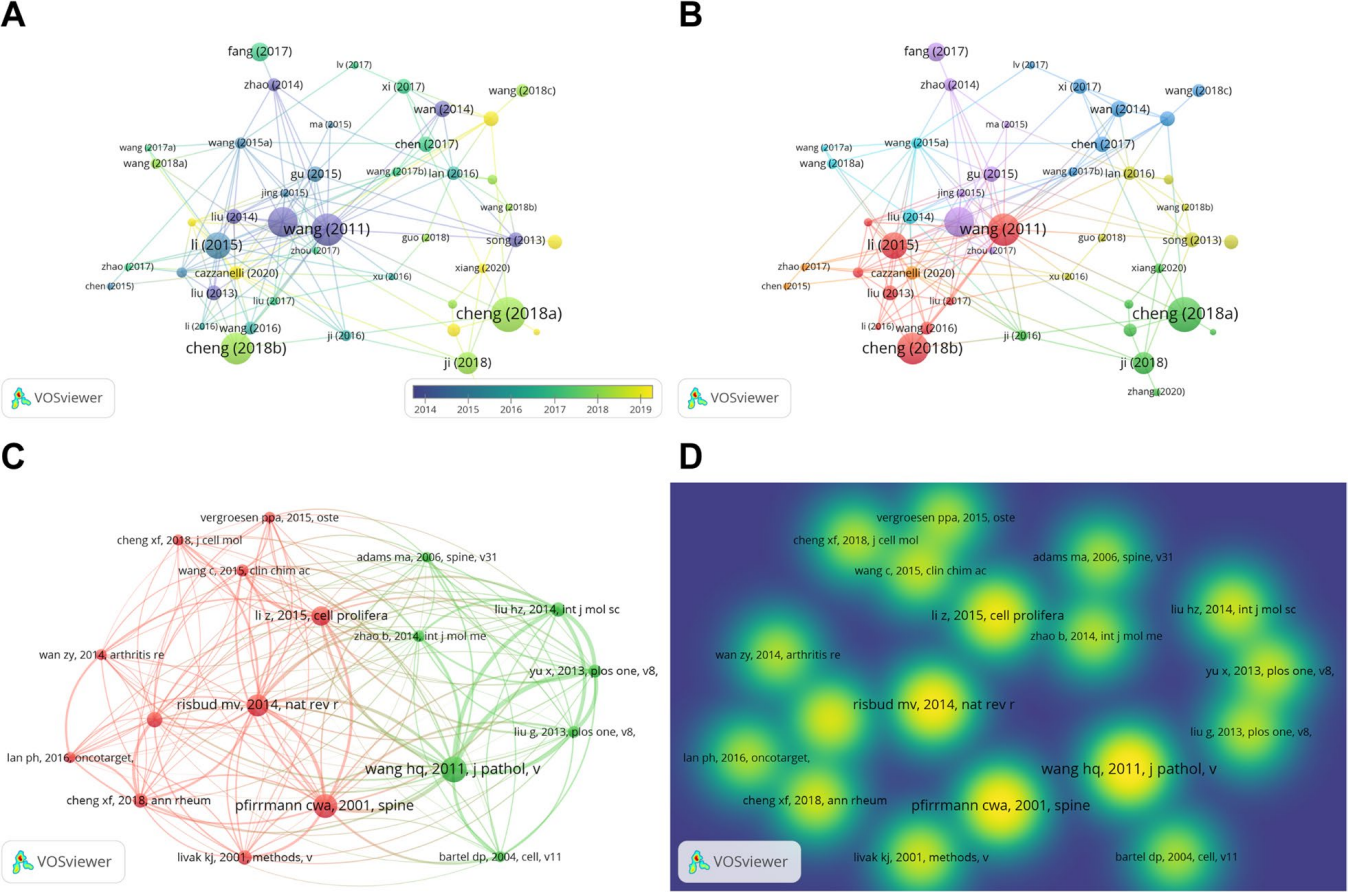
Characteristics of highly cited and co-citation articles on miRNAs and IDD. **A** Overlay visualization of highly cited documents. A node represents a highly cited document. The size of the node is directly proportional to the number of times it is cited. The cold or warm colours represent the time point of the literature published. Cheng (2018a), Wang (2011), and Cheng (2018b) ranked top three in terms of citations. Wang (2011) was the earliest research paper published. **B** Visual network map of cited articles. Different colours represent different clusters. **C** Visual network map of co-citation articles. Different colours represent different clusters. The node represents the co-citation article. The size of the node is directly proportional to the number of times the co-citation article was cited. **D** Visual density view of co-cited references. The yellow-green colour represents the co-citation article, whose density is positively correlated with the number of citations

Co-citation analysis is a research method to measure the degree of relationship between different documents, journals, or authors. The co-citation analysis found that 9198 references were generated from 337 articles. The most frequently cited reference was the first study in this field published by Wang HQ [22] in *The Journal of Pathology* in 2011, which also appeared in the highly cited articles in this field (Table 5). The VOSviewer software was used to draw the visualization maps of highly cited documents and co-cited documents. Only the documents with citation frequency ≥ 35 were shown in the plots for better visualization effect. Each node represented a document, and the node size represented the frequency of citation. The highly cited literature was divided into seven clusters (Fig. 6A, B), and Wang (2011), Cheng (2018a), and Li (2015) were grouped into cluster 1, whereas Cheng (2018b) was published later and belonged to cluster 2. The co-citation articles formed two clusters (Fig. 6C, D). Wang HQ (2011) with the most co-citations was clustered into cluster 1, and Pfirrmann CWA (2001) belonged to cluster 2.

**Table 5:** Top 10 co-citation articles on miRNAs and IDD in the WOSCC database

Rank	Author	Title	Journal	Year	Citations
1	Wang HQ	Deregulated miR-155 promotes Fas-mediated apoptosis in human intervertebral disc degeneration by targeting FADD and caspase-3	*The Journal of Pathology*	2011	85
2	Pfirrmann CWA	Magnetic resonance classification of lumbar intervertebral disc degeneration	*Spine*	2001	79
3	Risbud MV	Role of cytokines in intervertebral disc degeneration: pain and disc content	*Nature Reviews. Rheumatology*	2014	73
4	Li Z	MicroRNA in intervertebral disc degeneration	*Cell proliferation*	2015	65
5	Ji ML	Preclinical development of a microRNA-based therapy for intervertebral disc degeneration	*Nature Communications*	2018	52
6	Cheng X	Circular RNA VMA21 protects against intervertebral disc degeneration through targeting miR-200c and X-linked inhibitor-of-apoptosis protein	*Annals of the Rheumatic Diseases*	2018	48
7	Liu H	miR-21 promotes human nucleus pulposus cell proliferation through PTEN/AKT signalling	*International Journal of Molecular Sciences*	2014	47
8	Livak KJ	Analysis of relative gene expression data using real-time quantitative PCR and the 2(-Delta Delta C(T)) Method	*Methods*	2001	47
9	Yu X	MicroRNA-10b promotes nucleus pulposus cell proliferation through RhoC-Akt pathway by targeting HOXD10 in intervertebral disc degeneration	*PloS One*	2013	43
10	Wang C	MicroRNAs: New players in intervertebral disc degeneration	*Clinica Chimica Acta*	2015	42

*miRNAs* microRNAs; *IDD* Intervertebral disc degeneration; *WOSCC* Web of Science core collection

### Keyword analysis

We used VOSviewer to extract and cluster keywords for miRNAs studies involving IDD (Additional file 1: Table S2). The top five keywords in terms of occurrences were “intervertebral disc degeneration” (211), “expression” (144), “apoptosis” (135), “nucleus pulposus cells” (120), and “proliferation” (82).

**Fig. 7:**
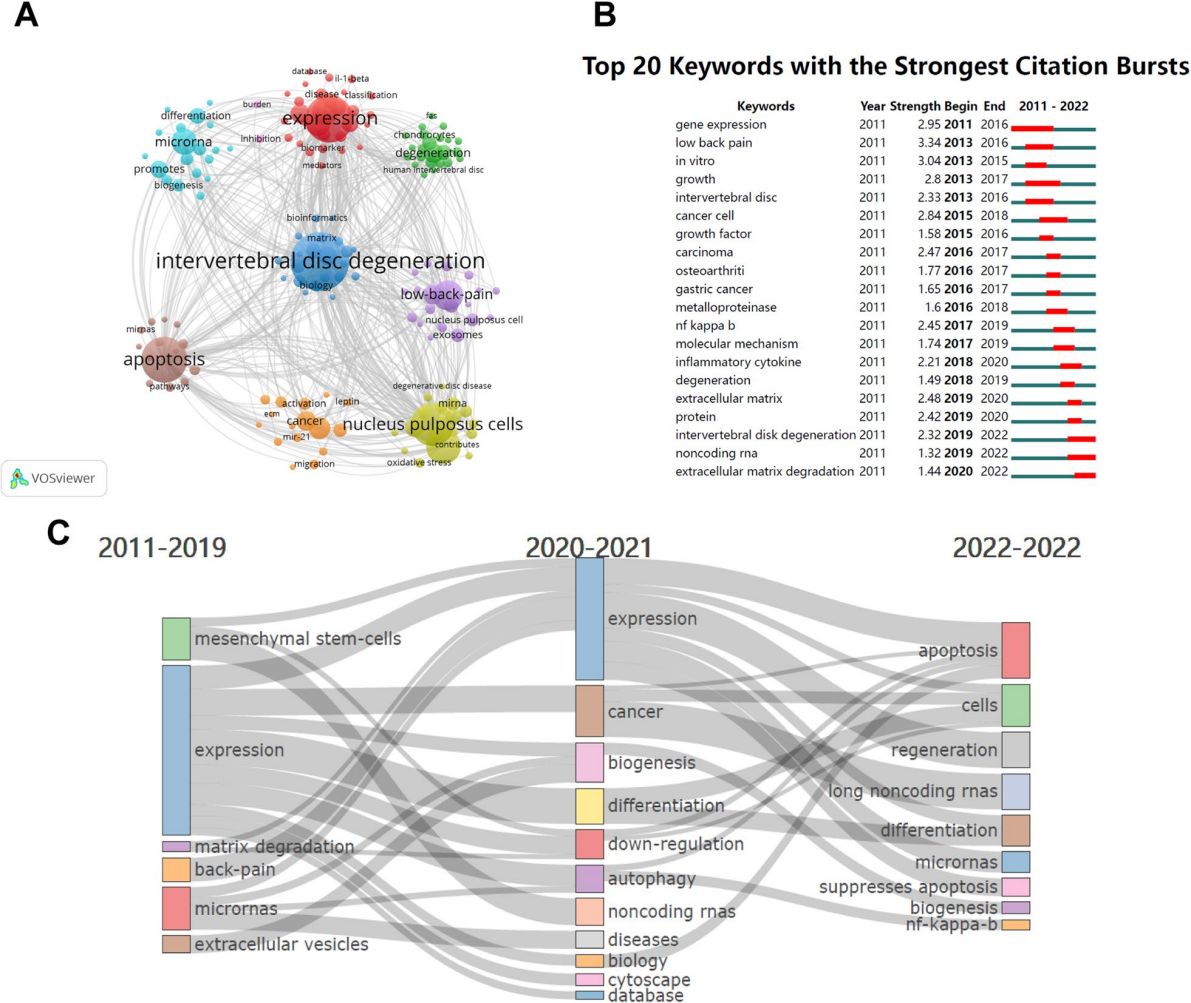
Keyword analysis of miRNAs and IDD research. **A** Keyword clustering network map. Nodes represent keywords, and larger nodes represent more keyword occurrences. Different colours represent different clustering clusters. **B** Top 20 Keywords with the strongest citation bursts in the WOSCC database. Red represents a high degree of keyword emergence in this period, whereas green represents a low degree of emergence. **C** Thematic evolution of keywords

We found that 166 keywords appeared more than four times, and these keywords formed eight clusters (Fig. 7A), including “intervertebral disc degeneration” (dark blue cluster), “apoptosis” (brown cluster), “cell-proliferation” (purple cluster), “nucleus pulposus cells” (yellow-green cluster), and “expression” (red cluster). The CiteSpace software can detect emergent keywords with high-frequency change rates and fast growth rates, and the emergent keywords can reflect the research frontiers in this field to a certain extent [25]. The results of the keyword emergence analysis (Fig. 7B) revealed that “gene expression”, “growth”, and “in vitro” were the most emergent keywords. In the last five years, “inflammatory cytokine”, “noncoding rna”, and “extracellular matrix degradation” were the emerging directions of research in this field. In addition, we found that the research themes changed over time with “apoptosis”, “long noncoding rnas”, “regeneration,” and “nf-kappa-b” being the most popular themes in 2022 (Fig. 7C).

### Analysis of potential mechanisms based on the Arrowsmith project

**Fig. 8:**
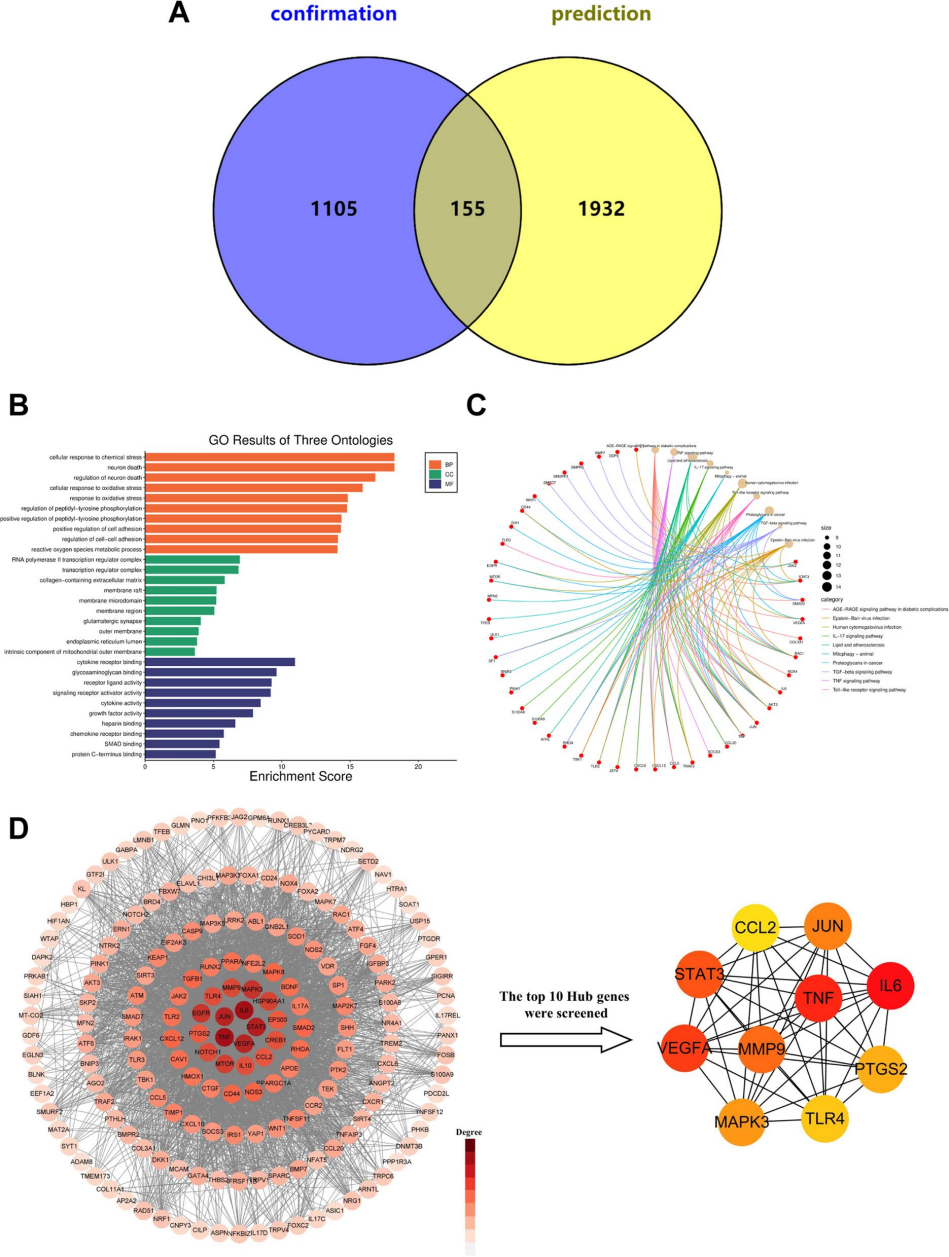
Potential keyword analysis of miRNAs and IDD research. **A** Venn diagrams for keywords of prediction and confirmation groups. **B** GO analysis of the potential key genes. **C** KEGG pathway enrichment analysis of the potential key genes. **D** PPI network and hub genes of miRNA-related targets in IDD

We entered the Arrowsmith project online search interface and set the search formula for A-query to [MicroRNAs (MeSH Terms)] and the search formula for C-query to [Intervertebral Disc Degeneration (MeSH Terms)]. The number of papers in Collection A was 25,000, and the number of papers in Collection C was 10,179. The Arrowsmith project was used to link Collection A and Collection C to form Collection B with 5151 associated words, which were then filtered by the word category filtering tool on the pages Activities & Behaviors, Anatomy, Chemicals & Drugs, Genes & Molecular Sequences, Gene & Protein Names, and Physiology to obtain 2087 keywords. These keywords were used as the prediction group, and the keywords extracted by VOSviewer were used as the confirmation group. Figure 8A shows the Venn diagram of the two groups. We obtained 1932 keywords as potential research topics for miRNAs and IDD, and the potential keywords with correlation probabilities ≥ 0.95 are shown in Additional file 1: Table S3. We extracted the genes of potential keywords for GO analysis and KEGG pathway enrichment analysis (Fig. 8B, C). The results of GO analysis revealed that biological processes (BP) were focused on cellular response to chemical stress, neuron death, regulation of neuron death, and cellular response to oxidative stress. Cellular components (CC) were mainly concentrated in RNA polymerase II transcription regulator complex and transcription regulator complex and collagen-containing extracellular matrix. Molecular functions (MF) were significantly enriched in potential genes in H1 receptor binding, glycosaminoglycan binding, and receptor–ligand activity. KEGG pathway enrichment analysis showed that the most significant pathways included TNF, IL-17, and TGF-beta signalling pathways. We further constructed a PPI network of these targets and screened for the hub genes and found that IL6, TNF, VEGFA, STAT3, and MMP9 were the most closely related potential targets of miRNAs and IDD, suggesting that the inflammatory response played a key role in IDD (Fig. 8D).

## Discussion

### General research trends on the role of miRNAs in IDD

We presented a bibliometric analysis of studies related to miRNAs and IDD in the WOSCC database to assess the current status, hot spots, and research trends in the field. According to the annual and annual cumulative publications, the research on miRNAs in IDD is an active research area. The results of the country/region contribution analysis showed that China had the highest number of publications and citations. The results of the country/region cooperation analysis suggested that China played a key role in this research area by collaborating worldwide. The institutions with the most publications were Huazhong University of Science and Technology, Shanghai Jiao Tong University, and Xi’an Jiao Tong University. However, the cooperative analysis suggested that the connections between the core institutions were not strong and should be strengthened through cooperation between institutions. The journal *Experimental and therapeutic medicine* had the most publications in this field. *Annals of the Rheumatic Diseases, The Journal of Pathology*, and *Nature Communications* were the most influential journals in this field. Therefore, the researchers who are exploring the role of miRNAs in IDD can prioritize these core journals. Wang K and Yang C had the highest number of publications as the core authors in the field, and Wang HQ had the highest number of citations. Interestingly, these three authors were from the Huazhong University of Science and Technology. Therefore, the scientists who are pursuing research in this area can learn from the experiences of the research team at the Huazhong University of Science and Technology and strengthen communication with these scholars. The amount of citations for articles depends on the time of publication; therefore, the time of publication has a significant effect on the citations of articles. Although an article may take at least 15 years to reach its peak number of citations [26], three articles were among the top 10 most cited articles in the last 5 years [21, 23, 24]. In the next few years, the number of citations of these articles may continue to increase, and these articles deserve our special attention. Based on the co-citation analysis, we found that the top 10 cited references included three reviews on the role of cytokines and immune cells in the catabolic process of IVD and miRNAs in regulating the function of NPCs in IDD.

### Hot spots and research trends on the role of miRNAs in IDD

We have summarized the research hotspots and development directions of the research on the role of miRNAs in IDD based on the results of keyword analysis and the potential mechanism analysis of the Arrowsmith project (Table 6).

**Table 6:** miRNAs involved in the process of IDD

MiRNA	Experimental models	Targets	Expression	Effect on IDD	References
*miRNAs involved in NPC apoptosis in IDD*					
miR-155	Human NPC	FADD, Caspase-3	Downregulated	Promotion	[22]
miR-15a	Human NP tissues	MAP3K9	Upregulated	Promotion	[27]
miR-573	Human NPC	Bax, Bcl-2, Caspase-3	Upregulated	Inhibition	[28]
miR-210	Human NP tissues	HOXA9	Downregulated	Inhibition	[29]
miR-499a-5p	Human NPC	SOX, TNF-α	Downregulated	Inhibition	[30]
*miRNAs involved in NPC proliferation in IDD*					
miR-10b	Human NP tissues	HOXD10, RhoC, Akt	Upregulated	Promotion	[31]
miR-21	Human NP tissues	PTEN, Akt	Upregulated	Promotion	[32]
miR-96	Human NPC	ARID2, Akt	Upregulated	Promotion	[33]
*miRNAs involved in the ECM degradation*					
miR-100	Human NP SV40 cell line	FGFR3	Upregulated	Promotion	[34]
miR-93	Human NP tissues	MMP-3	Downregulated	Promotion	[35]
miR-15b	Human NP tissues	SMAD3, IL-1β	Upregulated	Promotion	[36]
*miRNAs involved in the CEP degeneration*					
miR-34a	Human degenerative CEP tissues	Bcl-2, Fas	Upregulated	Promotion	[37]
miR-20a	Human degenerative CEP tissues	ANKH	Upregulated	Inhibition	[38]
miR-221	Human IDD CEP	ERα	Upregulated	Promotion	[39]
*miRNAs involved in inflammation in IDD*					
miR-181a	Rat NPC	TRAIL	Downregulated	Inhibition	[40]
miR-149	LPS-stimulated NPC	MMP3, ADAMTS, TNF-α, IL-1, IL-6	Downregulated	Inhibition	[41]
miR-15b	Human NP tissues	SMAD3, IL-1β	Upregulated	Promotion	[36]

*miRNAs* microRNAs; *IDD* Intervertebral disc degeneration; *NP* Nucleus pulposus; *NPC* NP cell; *IDD* Intervertebral disc degeneration; *ECM* Extracellular matrix; *FADD* Fas-associating protein with a novel death domain; *MAP3K9* Mitogen-activated protein 3 kinase 9; *Bcl-2* B-cell lymphoma-2; *Bax* BCL-2-associated X protein; *HOXA9* Homeobox A9; *SOX* Sex-determining region Y-box; *LPS* Lipopolysaccharide; *ERα* Estrogen receptor α

#### miRNAs involved in NPC apoptosis in IDD

Apoptosis, also known as type I programmed cell death, is characterized by chromosome condensation, cell shrinkage, DNA degradation, and apoptotic vesicle morphology. Apoptosis not only exists in various physiological processes but also extensively participates in the pathological processes of many degenerative diseases such as IDD and osteoarthritis [42]. Most miRNAs were highly expressed in the degenerated NPCs and promoted apoptosis. For example, miR-155 and miR-15a promoted apoptosis of NPCs through the Fas and MAPK pathways. Wang et al. [22] applied gene microarray technology to find 29 miRNA molecules with significant differential expression and found that miR-155 was significantly downregulated in NPCs. The authors found that overexpression of miR-155 in NPCs can promote NPC apoptosis by inhibiting the Fas-associated with death domain protein (FADD) and caspase-3 in the apoptosis-mediated pathway. Cai et al. [27] found that the expression of miR-15a was significantly upregulated in the degenerated NP tissues, and miR-15a was negatively correlated with the expression of MAP3K9 in NPCs. Therefore, miR-15a regulated NPC apoptosis by targeting MAP3K9.

In contrast, some authors have shown that miR-573, miR-210, and miR-499a-5p inhibited the process of NPC apoptosis [28–30]. Wang et al. [28] found that miR-573 was overexpressed in the degenerative NPCs, which acted on the pro-apoptotic protein Bax to downregulate its expression. Subsequently, the expression of anti-apoptotic protein Bcl-2 was increased, whereas the expression of apoptotic protease caspase-3 was decreased, thereby inhibiting NPC apoptosis. Zhang et al. [29] detected the expression of miR-210 in the NP tissues of 12 patients with scoliosis by reverse-transcription-quantitative polymerase chain reaction (qPCR) and found that the expression of miR-210 in the NP tissues of the degenerative IVD group decreased significantly. The authors suggested that the low expression of miR-210 inhibited the expression of target protein HOXA9, regulated the apoptosis of NPCs, and inhibited the development of IDD. Sun et al. [30] discovered that miR-499a-5p expression was decreased in the regressed group compared to the healthy human NPC tissues, and miR-499a-5p inhibited TNF-α to delay NPC apoptosis through its direct target sex-determining region Y-box (SOX4). The above studies indicated that miRNAs directly or indirectly promoted NPC apoptosis and affected the pathological process of IDD through related pathways.

#### miRNAs involved in NPC proliferation in IDD

One of the pathological features of IDD is the abnormal proliferation of NPCs and the formation of cell clusters, which represent the self-repairing ability of IVDs. The abnormal proliferation of NPCs is closely related to the progress of IDD [43, 44]. Yu et al. [31] found that miR-10b, whose expression was significantly elevated in the degenerated disc tissues, could play a role in the pathology of disc degeneration by mediating abnormal NPC proliferation through the regulation of the RhoC–Akt pathway. Liu et al. [32] found that the miR-21 expression was significantly elevated in the NP tissues of degenerated discs. Further studies revealed that miR-21 promoted abnormal NPC proliferation by downregulating its target gene PTEN and increasing Akt phosphorylation, which, in turn, influenced the PTEN–Akt-mediated signal transduction pathway. Tao et al. [33] used reverse transcription-qPCR to detect the miR-96 expression in NP tissues from patients with IDD and healthy tissues from patients with traumatic lumbar fractures. The authors used dual luciferase analysis and confirmed that AT-rich interaction domain 2 (ARID2) was the direct target gene of miR-96. The results suggested that miR-96 may activate the Akt pathway phosphorylation by targeting ARID2 to promote the proliferation of human degenerated NPCs, which may become a therapeutic target for IDD. However, the mechanisms of abnormal proliferation remain unclear and further research is required in this context.

#### miRNAs involved in the ECM degradation

ECM is always in the dynamic balance of the interaction between NPC synthesis and matrix degradation in normal IVDs. In addition, ECM maintains the integrity of IVD and regulates the survival, morphology, and differentiation of cells in the disc tissues. However, ECM degradation/synthesis is imbalanced in the degenerated disc tissues leading to a decrease in the ECM components, such as collagen and proteoglycans, in the NP tissues, which eventually causes a series of pathological changes such as loss of disc height [45]. Matrix metalloproteinases (MMPs) and a disintegrin-like metalloproteinase with thrombospondin type-1 motifs (ADAMTS) are the main proteases that degrade the components of ECM. Yan et al. [34] revealed that miR-100 inhibited the translation of the FGFR3 mRNA by targeting its 3′-UTR, thereby activating MMP-13 and promoting ECM degradation in human NPCs. Jing et al. [35] discovered that the expression of miR-93 was significantly downregulated in the degenerated NP tissues in humans, and its expression was negatively correlated with the degree of IDD. The authors further revealed that the overexpression of miR-93 targeted and silenced MMP-3 to stimulate the expression of type II collagen in degenerated NPCs, thereby suggesting that the low expression of miR-93 may be associated with the increase in matrix-degrading enzymes and the change in ECM components in IDD.

In addition to mediating the expression of MMP, miRNA can also interfere in the pathological process of IDD by regulating the expression of ADAMTS. Kang et al. [36] reported that miR-15b was upregulated in NP tissues with disc degeneration and in NPCs stimulated with IL-1β compared with normal controls. Overexpression of miR-5b suppressed its expression by targeting its binding to SMAD3 and upregulated the levels of ADAMTS4, ADAMTS5, MMP3, and MMP13 protein in the IL-1β-induced NPCs. This indicated that the expression of matrix enzymes mediated by inflammatory factors could be inhibited or downregulated by suppressing miR-15b expression, thereby protecting the ECM of IVDs. Overall, these studies suggested that miRNAs mediated the synthesis and degradation of ECM of IVDs by regulating MMPs and ADAMTS, thereby delaying the development of IDD. These miRNAs regulate the homeostasis of ECM by regulating the corresponding target genes and finally acting on the MMPs and ADAMTS proteins. However, most of these studies were limited to the expression levels, and the correlation and the cascade of the regulation process were not clear; therefore, further experimental evidence is required to design miRNA-based therapeutic strategies.

#### miRNAs involved in the CEP degeneration

The cartilage endplate (CEP) is a natural barrier to prevent inflammatory injury mediators from entering the discs. Calcification of the endplate can lead to diffusion dysfunction, affect substance exchange, destabilize the IVD, and cause/accelerate IDD. miRNAs are closely related to CEP cell apoptosis. Chen et al. [37] found that the miR-34a expression and apoptotic cell counts were notably increased in the degenerative CEP cells, and miR-34a promoted Fas-mediated apoptosis by targeting Bcl-2, which accelerated the development of IDD. Liu et al. [38] found that miR-20a was significantly upregulated and the progressive ankylosing protein was downregulated with an increase in the matrix hardness of CEP, which indicated that these molecules were involved in the potential regulatory pathways that inhibit the degeneration of CEP. Shen et al. [39] used qPCR to find that the expression level of miR-221 was significantly upregulated in the degenerative CEP cells and identified the estrogen receptor α (ERα) gene as its target gene by transfection with a luciferase reporter plasmid, thereby confirming that the upregulation of miR-221 weakened the protective effects on the discs by acting on the 17beta-estradiol (E2) and leading to endplate apoptotic cell death. Therefore, genetic engineering approaches to normalize the expression of the dysregulated miRNAs may delay the progression of IDD by mediating apoptosis in the CEP. However, few relevant studies are available at present, and further detailed studies are required.

#### miRNAs involved in inflammation in IDD

Although the mechanisms of IDD are still unclear, several authors have reported that inflammation plays a key role in the occurrence of IDD. Inflammatory mediators (such as IL-1, IL-6, and TNF-α), apoptosis, cell senescence, and oxidative stress cause inflammation and promote the development of IDD. Sun et al. [40] treated mouse NPCs with miR-181a and found a marked decrease in the expressions of TNF-α, TGF-β1, IL-6, and IL-13. Moreover, they confirmed that the upregulation of miR-181a alleviated the inflammatory reaction and delayed the development of IDD by inhibiting TNF-related apoptosis-inducing ligands. Qin et al. [41] also showed that miR-149 inhibited the TLR4 signalling pathway by targeting the MyD88 protein and inflammatory cytokine (including TNF-α, IL-1, and IL-6) production induced by lipopolysaccharides, which, in turn, decreased ECM degradation. Kang et al. [36] discovered that the expression of miR-15b was upregulated in degenerated IVD tissues using qPCR. Moreover, the stimulation of NPCs with IL-1β also upregulated miR-15b. Kang et al. cotransfected miR-15b inhibitor and siSMAD3 and found that the highly expressed miR-15b acted on SMAD3 and enhanced the degradation of ECM mediated by IL-1β, whereas the inhibition of miR-15b had the opposite effect. Taken together, these studies indicated that miRNAs play an important role in the inflammatory mechanism of IDD through different pathways, thereby suggesting a new therapeutic approach for IDD.

#### Prospects of therapeutic applications of miRNAs in DDD

**Fig. 9:**
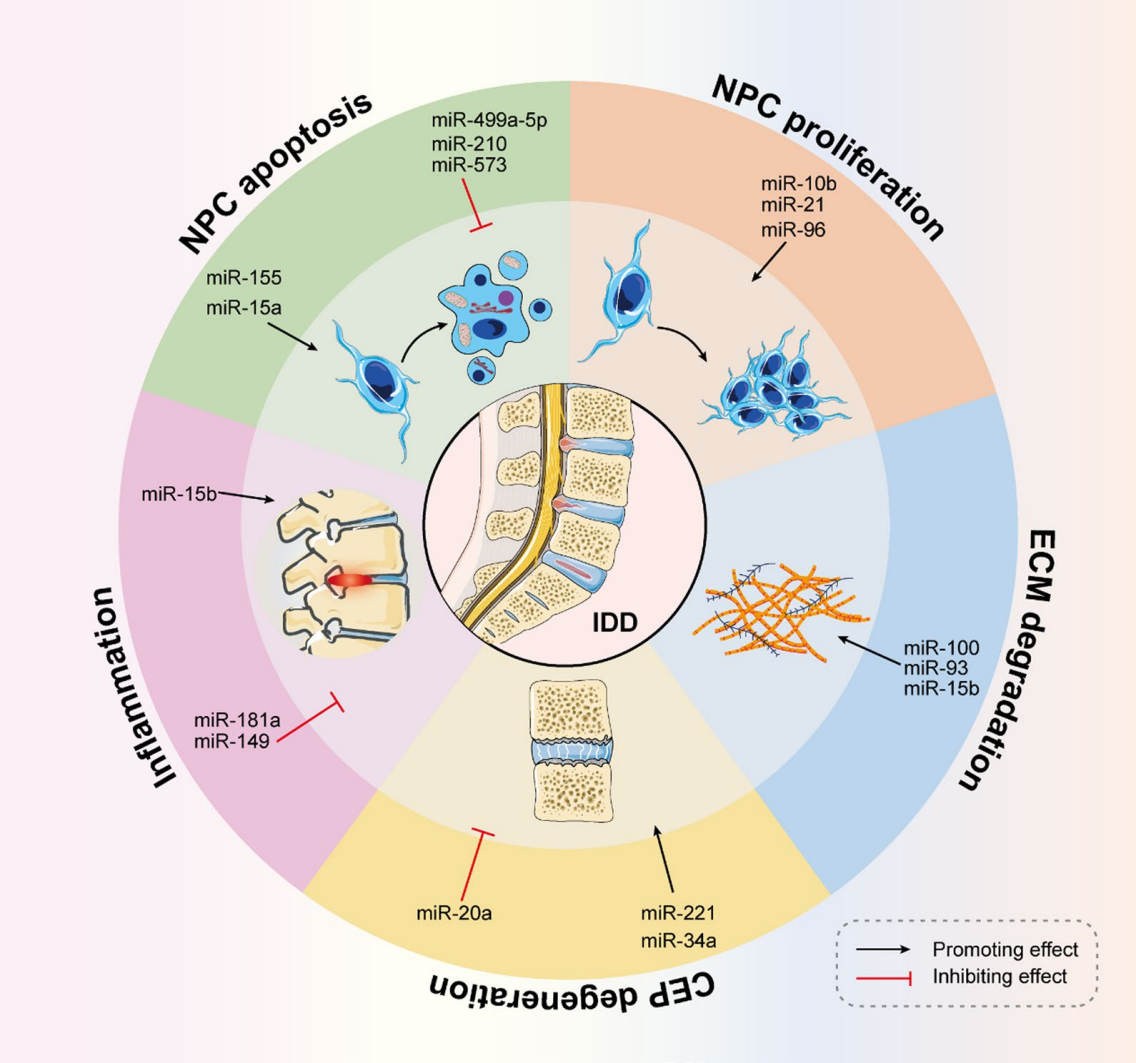
miRNAs involved in the pathogenesis of IDD

In the last decade, the research focus has shifted from screening for disease-associated miRNAs to in-depth studies on the role of miRNAs in disease development and potential miRNA-based therapeutic applications. MiRNAs are involved in multiple pathological processes in the development of IDD (Fig. 9); however, there are many challenges in the therapeutic application of miRNAs in IDD. First, current research on miRNAs involved in IDD is limited to NP and CEP cells, and the effects of miRNAs on the annulus fibrosus cells have not been clarified. Second, oxidative stress, cell ageing, and autophagy play key roles in IDD. It is unclear whether miRNAs also affect these pathological processes. Third, only in vitro studies have been conducted to explore the role of miRNAs in IDD pathogenesis, and extensive research involving animal models and clinical patients is not available. Finally, the dosage of miRNA therapy remains unclear, and further studies are needed to avoid adverse reactions to miRNA therapy.

### Strengths and limitations

This is the first bibliometric analysis of studies related to miRNAs and IDD extracted from the WOSCC database to identify new strategies for the prevention and treatment of IDD. We also revealed the potential mechanisms by which miRNAs were involved in the IDD pathogenesis based on the Arrowsmith project. However, our study had some limitations. We only retrieved the literature from the WOSCC database and did not include the literature in other databases, which may lead to the literature omission and bias. In addition, recently published high-quality literature may have gone unnoticed because of the lower citations. Future studies should comprehensively search the literature in related fields in the database and conduct more detailed comparative research and analysis.

## Conclusion

We demonstrated the knowledge map of miRNAs and IDD-related research in terms of countries, institutions, journals, authors, and cited literature through bibliometric analysis, determined the current research status and hotspots in this field, and constructed the potential mechanisms of the involvement of miRNAs in IDD using the Arrowsmith project. We found that the number of miRNAs and IDD-related publications showed an overall increasing trend, and it is an active area of research. China was the most important contributor to research in this field. The top three institutions in terms of the number of articles published were Huazhong University of Science and Technology, Shanghai Jiao Tong University, and Xi’an Jiao Tong University. Shanghai Jiao Tong University had the highest number of citations. The journal *Experimental and thermal medicine* had the most documents, and *Cell promotion* had the most citations. The journal with the most mean times cited per study was *Annals of the Rheumatic Diseases*. These were the authoritative journals in this field. The author with the most publications was Wang K, and the author with the most citations was Wang HQ. These two authors have made important contributions to research in this field. Keyword co-occurrence, clustering, and emergent analysis showed that the recent studies in this field have focused on miRNAs regulating apoptosis and proliferation of degenerative disc cells. In addition, we determined the potential mechanisms of the involvement of miRNAs in IDD, including miRNAs regulating the ECM degradation, mediating CEP degeneration, and participating in inflammatory responses. These mechanisms should be explored further to design optimal therapeutic strategies that target miRNAs in IDD.

## Supplementary Information

**Additional file 1: Fig. S1.** Flow chart of search strategy and document screening. **Table S1.** Top 10 journals in terms of number of documents and citations on miRNAs in IDD. **Table S2.** Top 20 keywords on miRNAs in IDD. **Table S3.** The potential keywords of miRNAs and IDD research.

## Data Availability

The datasets used and analysed in the current study are available from the corresponding author on reasonable request.
