# Supplementary material for: Research hotspots and trends of microRNAs in intervertebral disc degeneration: a comprehensive bibliometric analysis
Source: J Orthop Surg Res. 2023 Apr 15;18:302. doi: 10.1186/s13018-023-03788-4 (PMC10105931; doi:10.1186/s13018-023-03788-4)
Supplement: Supplementary file 1 — Additional file 1: Fig. S1. Flow chart of search strategy and document screening. Table S1. Top 10 journals in terms of number of documents and citations on miRNAs in IDD. Table S2. Top 20 keywords on miRNAs in IDD. Table S3. The potential keywords of miRNAs and IDD research. [file 13018_2023_3788_MOESM1_ESM.docx]

**Supplementary Figures and Tables**

**Supplemental Figure**


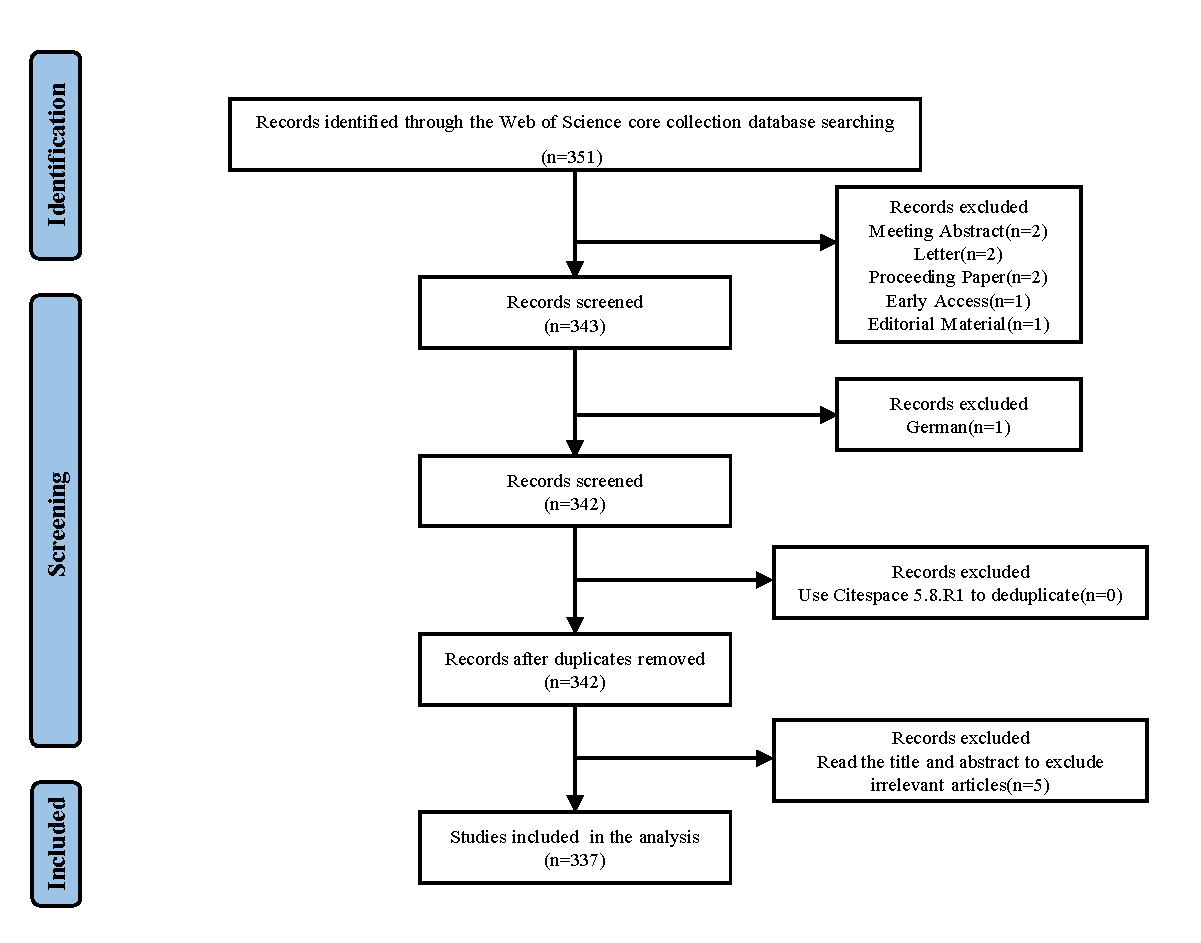


**Supplemental Figure 1** Flow chart of search strategy and document screening.

**Supplemental Tables**

**Supplemental Table S1** Top 10 journals in terms of number of documents and citations on miRNAs in IDD

| Rank | Journal (n=151) | Documents (%) | JCR partition/IF | Mean times cited per study | Rank | Journal (n=151) | Citations (%) | JCR partition/IF | Mean times cited per study^✝^ |
| --- | --- | --- | --- | --- | --- | --- | --- | --- | --- |
| 1 | *Experimental and therapeutic medicine* | 18 (5.34) | Q4 (0.22) | 6.61 | 1 | *Cell proliferation* | 302 (5.12) | Q2 (3.85) | 50.33 |
| 2 | *Molecular medicine reports* | 16 (4.75) | Q4 (0.22) | 14.50 | 2 | *Journal of cellular and molecular medicine* | 272 (4.61) | Q3 (4.06) | 38.86 |
| 3 | *Frontiers in cell and developmental biology* | 9 (2.67) | Q2 (3.85) | 6.67 | 3 | *Plos one* | 254 (4.31) | Q3 (4.06) | 84.67 |
| 4 | *Oxidative medicine and cellular longevity* | 8 (2.37) | Q3 (4.06) | 1.88 | 4 | *Molecular medicine reports* | 232 (3.93) | Q4 (0.22) | 14.50 |
| 5 | *Journal of cellular and molecular medicine* | 7 (2.08) | Q3 (4.06) | 38.86 | 5 | *Biomedicine & pharmacotherapy* | 217 (3.68) | Q2 (3.85) | 31.00 |
| 6 | *Biomedicine & pharmacotherapy* | 7 (2.08) | Q2 (3.85) | 31.00 | 6 | *Annals of the rheumatic diseases* | 176 (2.98) | Q1 (16.81) | 176.00 |
| 7 | *Cell cycle* | 7 (2.08) | Q4 (0.22) | 11.14 | 7 | *Journal of pathology* | 163 (2.76) | Q1 (16.81) | 163.00 |
| 8 | *Biomed research international* | 7 (2.08) | Q3 (4.06) | 6.43 | 8 | *Gene* | 161 (2.73) | Q3 (4.06) | 40.25 |
| 9 | *Cell proliferation* | 6 (1.78) | Q2 (3.85) | 50.33 | 9 | *Journal of cellular biochemistry* | 159 (2.69) | Q3 (4.06) | 26.50 |
| 10 | *Journal of cellular biochemistry* | 6 (1.78) | Q3 (4.06) | 26.50 | 10 | *Journal of cellular physiology* | 152 (2.58) | Q2 (3.85) | 30.40 |

✝, the average number of citations per study in the journal. IF, impact factor. JCR, Journal Ranking by Clarivate Analytics Journal Citation Reports. miRNAs, microRNAs. IDD, intervertebral disc degeneration.

**Supplemental Table S2** Top 20 keywords on miRNAs in IDD

| Rank | Keyword | Occurrences | Total link strength | Rank | Keyword | Occurrences | Total link strength |
| --- | --- | --- | --- | --- | --- | --- | --- |
| 1 | Intervertebral disc degeneration | 211 | 1414 | 11 | Cancer | 32 | 241 |
| 2 | Expression | 144 | 1070 | 12 | Degeneration | 32 | 229 |
| 3 | Apoptosis | 135 | 977 | 13 | Inflammation | 31 | 234 |
| 4 | Nucleus pulposus cells | 120 | 881 | 14 | Autophagy | 30 | 207 |
| 5 | Proliferation | 82 | 598 | 15 | TNF-alpha | 30 | 267 |
| 6 | Low-back-pain | 65 | 508 | 16 | Cells | 29 | 187 |
| 7 | Microrna | 50 | 396 | 17 | Promotes | 26 | 211 |
| 8 | Nucleus pulposus | 50 | 379 | 18 | Intervertebral disc | 23 | 146 |
| 9 | Micrornas | 46 | 363 | 19 | Down-regulation | 22 | 199 |
| 10 | Pathway | 38 | 274 | 20 | Degradation | 21 | 153 |

miRNAs, microRNAs. IDD, intervertebral disc degeneration.

**Supplemental Table S3** The potential keywords of miRNAs and IDD research

| Rank | Correlation probabilities | Target/gene/protein | Pathway/mechanism | Treatment | | |
| --- | --- | --- | --- | --- | --- | --- |
| 1 | 0.99 | RANKL, JNK, JAK2, MTOR, CCL20, STAT3, ERK1, SMAD, PINK1, KEAP1, YAP1, RUNX1, CCN2, BNIP3, Toll receptor TLR4, COX-2, RUNX2, SMAD2, WTAP, SP1, SIRT3, SIRTUIN, CTGF, VEGFA, PARKIN, TLR2, TAK1, BDNF,T RPV4, LRRK2, FOXA2, SMAD7, Caspase-9, SOCS3, HIF-1, FOXA1, AMPK, ULK1, MEK, CREB, TBK1, COX2, SHH, NFAT5, FOXO, PGC-1alpha, TFEB, NRF2, HUR, IL-10, MMP-9, Vitamin d, Wnt beta catenin receptor, ASPORIN, KLOTHO, DNMT3B, PHD3, HTRA1, BRD4, FGF4, SMURF2, BMAL1, FBW7, TRAF2, CD44, FOXC2, COL3A1, RAC1, PDGF, caspase, TGFBETA, Hypoxia inducible factor-1, SKP2, CAVEOLIN-1, RNA binding protein, FDG, IRE1, APOE, RHOA, IRS-1, TRPM7, Cyclooxygenase-2, MFN2, CXCR1, KINASE B, NOX4, DRP1, S100A9, Interleukin-17, IL-22, TREM2, NR4A1, FAK, SIAH1, CD24, TWEAK, P70S6K, MAT2A, CCL5, S100A8, SOD1, TLR3, COL11A1, IL6, TGF BETA1, NRG1, WNT1, CYCLIN, TOLL, TNFALPHA, HSP90, PPAR | Beta catenin pathway, NF kb pathway, NOTCH signaling pathway, P38 mapk pathway, MAPK signaling pathway, TGF beta pathway, Apoptotic pathway, ERK signaling pathway, Autophagy pathway, Mitochondrial apoptotic pathway/apoptosis autophagy, apoptosis extracellular matrix, autophagy apoptosis, oxidative stress apoptosis | | Artificial intelligence, superparamagnetic iron oxide, chitosan hydrogel, tissue engineered |  |
| 2 | 0.98 | NFAT, IGFBP3, VNTR, MCP-1, FLT-1, CXCL10, PROTEIN-2, STATIN, ASK1, BMPR2, AKT3, KAPPA B, NAV1, TIMP1, ASIC1, NF KB, IL-17, TLR, Cell derived factor-1, INOS, CXCL6, STAT, STING, GATA4, IRAK-1, SPARC, TRKB, OPG, Angiopoietin-2, NOTCH2, P300, NARINGIN, CD146 | Apoptosis pathway, Kappab signal pathway, MAPK signalling pathway, P38-mapk pathway/migration extracellular matrix, apoptosis oxidative stress | | Embryonic stem cell, oxygen ozone therapy |  |
| 3 | 0.97 | MCAM, RNAI, NRF1, Heme oxygenase-1, VDR, NOTCH, TGF, P65, ATF4, DKK-1, A20, JUN, M6A, NTRK2, THBS2, HBP1, PFKFB3, TRPC6, PTHRP, ZIC5, Pannexin | Kinase pathway, senescence associated, endothelial cell migration, induced angiogenesis | | Platelet rich plasma, apigenin |  |
| 4 | 0.96 | TRPV1, AS1, BMP-7, ADAM8, ERK5, EGFR, BMP7, ATM, TNF, YKL-40, GDF6, ABL1, CCR2, CRE, HO-1 | Cell death pathway | | Panax notoginseng saponin, ginsenoside rd, stem cell based |  |
| 5 | 0.95 | LAMIN, GPR30, AP-1, ATF6, JAG2, C FOS, NDRG2, IL-21, RACK1, TIE2, NRF-2, NF, RAD51, RG1, ENOS, ANKH, PERK | Stress pathway, mitochondrial dysfunction oxidative | | Autologous bone marrow, inhibit tnf alpha |  |

miRNAs, microRNAs. IDD, intervertebral disc degeneration.
